# Gold-standard experiments to deter predators from attacking farm animals

**DOI:** 10.1093/af/vfad072

**Published:** 2024-02-14

**Authors:** A Treves, A R Fergus, S J Hermanstorfer, N X Louchouarn, O Ohrens, A Pineda-Guerrero

**Affiliations:** Nelson Institute for Environmental Studies, Carnivore Coexistence Lab, 30A Science Hall Science Hall, Madison, WI 53706, USA; Nelson Institute for Environmental Studies, Carnivore Coexistence Lab, 30A Science Hall Science Hall, Madison, WI 53706, USA; Nelson Institute for Environmental Studies, Carnivore Coexistence Lab, 30A Science Hall Science Hall, Madison, WI 53706, USA; Nelson Institute for Environmental Studies, Carnivore Coexistence Lab, 30A Science Hall Science Hall, Madison, WI 53706, USA; Panthera, New York, NY 10018, USA; Nelson Institute for Environmental Studies, Carnivore Coexistence Lab, 30A Science Hall Science Hall, Madison, WI 53706, USA

**Keywords:** carnivore, crossover-design experiments, domestic animals, livestock, predators, RCTs, randomization

**Implications**

- We dismiss the long-held belief that randomized, controlled trials (RCTs) are impossible in wild ecosystems with working livestock.
- Crossover designs reduce confounding variables between subjects and strengthen inference beyond the RCT, yet obstacles exist, which we describe qualitatively.
- Non-lethal methods can be effective in preventing carnivore approaches and attacks on farm animals in fenced pastures or open rangelands. The relationship between approaches and attacks remains uncertain.
- Lethal methods of predator control have been subjected to less robust study designs that suggest mixed results including increases in livestock losses. Non-lethals promise the elusive triple-win for wildlife, domestic animals, and livelihoods.

## Introduction

Climate change and extinctions now pose the major threats to life on our planet (Ripple et al., 2017; Chapron et al., 2018; Ceballos et al., 2020). Humans cause many extinctions by killing animals or by transforming habitats. Among mammals, large carnivores have faced higher than average rates of population extirpation because of direct and indirect competition with humans (Woodroffe and Ginsberg, 1998; Chapron et al., 2014; Ripple et al., 2014).

Humans respond to real and perceived threats from carnivores with lethal action and with sociopolitical pressure against protecting the last remaining carnivores. Therefore, interest groups and individuals focused on preserving carnivore populations and minimizing harm to individual carnivores have prioritized non-lethal methods to prevent conflicts between humans and carnivores in recent years. In addition to reducing damage to human property by carnivores, non-lethal methods offer potential benefits to many actors involved, by saving animal lives and benefiting human health, safety, and income. Here we describe lessons learned from gold-standard, randomized, controlled trials (RCTs) with crossover designs, which we have conducted in four countries to protect farm animals from wild carnivores of many species. We synthesize lessons learned in four categories: experiences with randomized, controlled trials (RCTs), design recommendations, effectiveness of non-lethal methods to prevent wild carnivore predation on farm animals, and conclusions. We place these in a global context with similar trials. We discuss gaps in evidence that should motivate investments in research and precautions among decision-makers at all levels.

## Experiences With RCTs

Inspired by experiments in the United Kingdom on badgers (*Meles meles*) to evaluate the effect of two interventions on transmission of bovine tuberculosis to cattle (Donnelly et al., 2003) and by Australian experimenters killing red fox (*Vulpes vulpes*) with poison, to evaluate the effect on predation of sheep (Greentree et al., 2000), we conducted our first predator-control RCT in Wisconsin, United States (Shivik et al., 2003). This first effort did not involve domestic animals (defined as commercial or subsistence, not captive colonies), hereafter farm animals or livestock. Still, this initial study proved the feasibility of robust RCT designs under field conditions, including crossover. Crossover occurs when treatment and control are reversed midway through a study so each subject experiences each condition. In the interim period before our next RCT, other studies proved the utility of randomized experiments to examine the effectiveness of non-lethal methods to influence wild, medium- to large-bodied carnivores preying on livestock (Bromley and Gese, 2001; Davidson-Nelson and Gehring, 2010; Gehring et al., 2010). Also, numerous experiments on American black bears (*Ursus americanus*) damaging non-mobile property suggested a need for RCTs with predators of livestock. Despite an RCT by Greentree et al. (2000) on fox poisoning in Australia, to date, no further reliable, peer-reviewed RCTs have been conducted on lethal intervention against predators of farm animals. See web panel 1 in Treves et al. (2016) for discussions of other quasi- or apparently experimental work that was considered unreliable because of design flaws or statistical biases. Greentree et al. (2000) concluded that there was no significant effect of poisoning red foxes and concluded much effort was wasted. It remains unclear if insights gained from the experimental killing of non-native carnivores preying on sheep in Australia can be generalized to native carnivores elsewhere. We raise this question because these and other non-natives are descendants of animals held in captivity by humans, which might affect their attraction to or fear of, humans in ways that modify the effectiveness of predator control. Our crossover RCTs build upon global research on non-lethal methods that used less robust designs (Stone et al., 2017).

**Figure 1.**
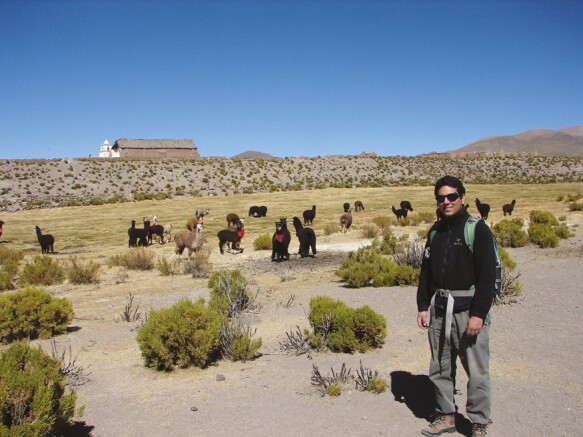
Dr. Omar Ohrens at study site, Tarapaca, Chile with alpacas. Credit A. Treves.

**Figure 2.**
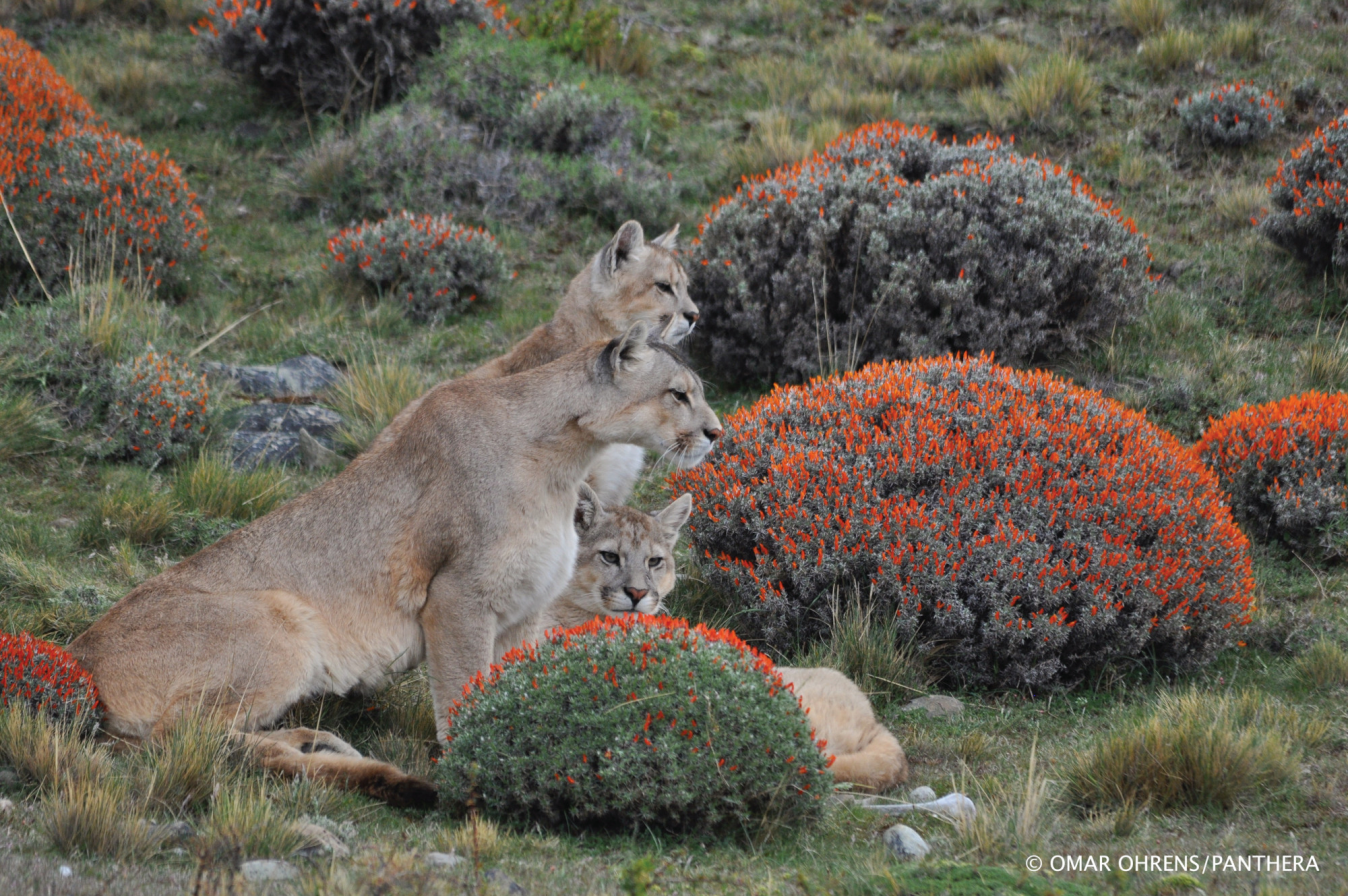
Puma in Chile. Credit O. Ohrens.

The second RCT by members of our group focused on large carnivores interacting with working llamas, alpacas, and sheep in Chile’s remote Andean altiplano (Figure 1). This study built on Ohrens et al. (2015) previous work in the region interviewing domestic animal owners by using participatory intervention planning methods for an RCT, citations in TrevesWallace and White, (2009) and Ohrens et al., (2019a). The recruited landowners participated by choosing to evaluate the non-lethal deterrent Foxlights, a commercially available random light projector triggered by nightfall (Ohrens et al., 2019a). Ohrens published his PhD dissertation as our first RCT with crossover design on working farm animals, aimed at protecting 11 herds of alpacas and llamas from pumas (*Puma concolor*), also known as cougar or mountain lion, using a light deterrent (Figure 2). His study paved the way for the next 4, including efforts at replication, while it also revealed the surprising attraction of Andean foxes (*Lycalopex* culpaeus) that we describe below, including an insight from his second experiment not yet published.

**Figure 3.**
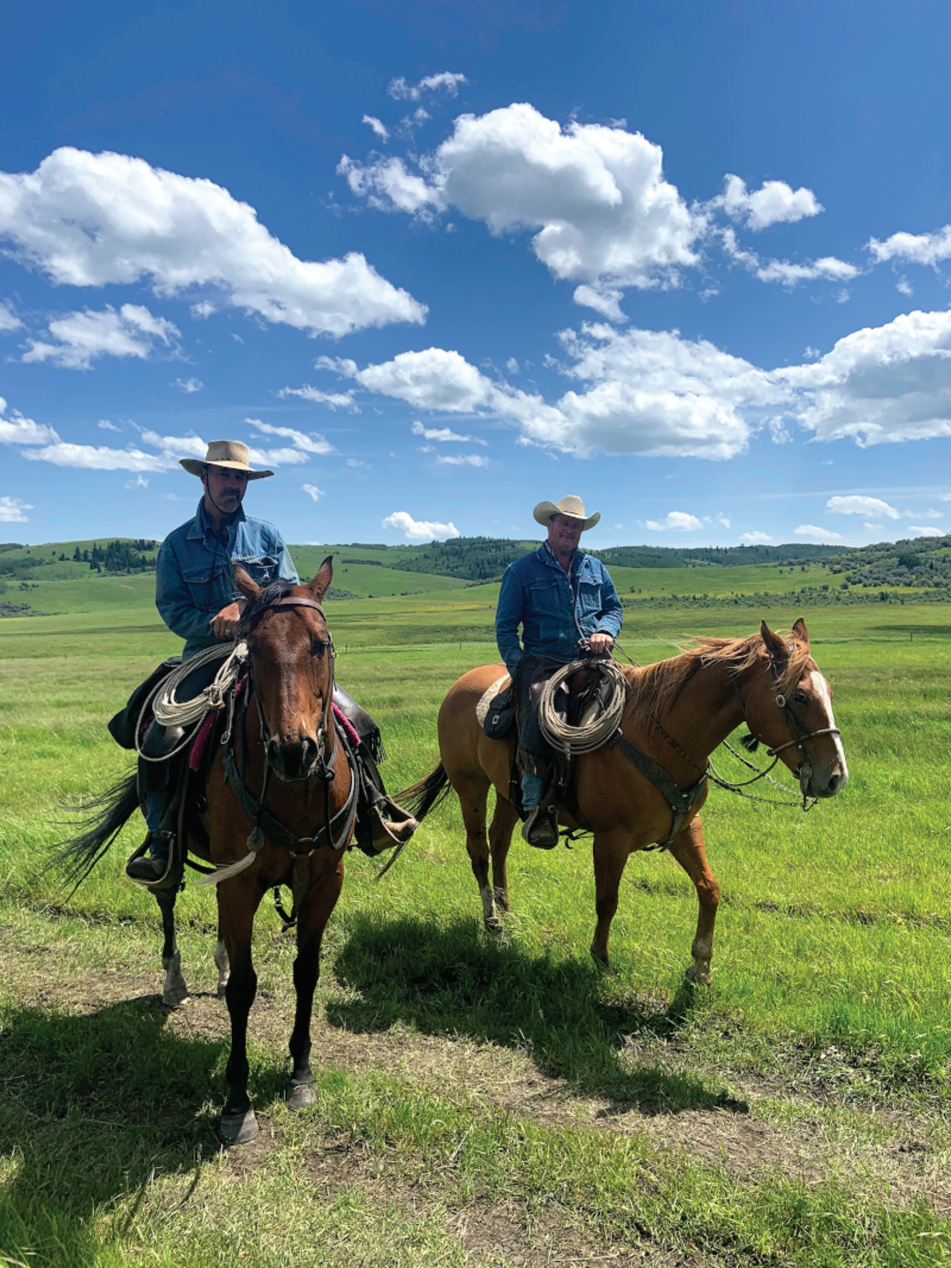
Range riders in Alberta. Credit. N. X. Louchouarn.

The second peer-reviewed RCT aimed to evaluate herders using low-stress livestock handling methods, citations in Louchouarn and Treves (2023)—hereafter range riders (Figure 3). Range riders aimed to protect cattle from brown (grizzly) bears, gray wolves (*C. lupus*), pumas, black bears, and coyotes (*C. latrans*) in the Canadian Rockies of Alberta. This method aims to enhance and promote natural herding and antipredator behavior in cattle. This study incidentally shed light on the use of a pseudo-control rather than placebo control. Louchouarn’s work also sheds the most light on any previous work on the design of range rider interventions (Louchouarn and Treves, 2023). Namely, that a single experienced range rider could deter large carnivores as effectively as several range riders with less experience. Nevertheless, the frequency of range rider visits (dose effect) seemed important to understanding range rider effectiveness.

**Figure 4.**
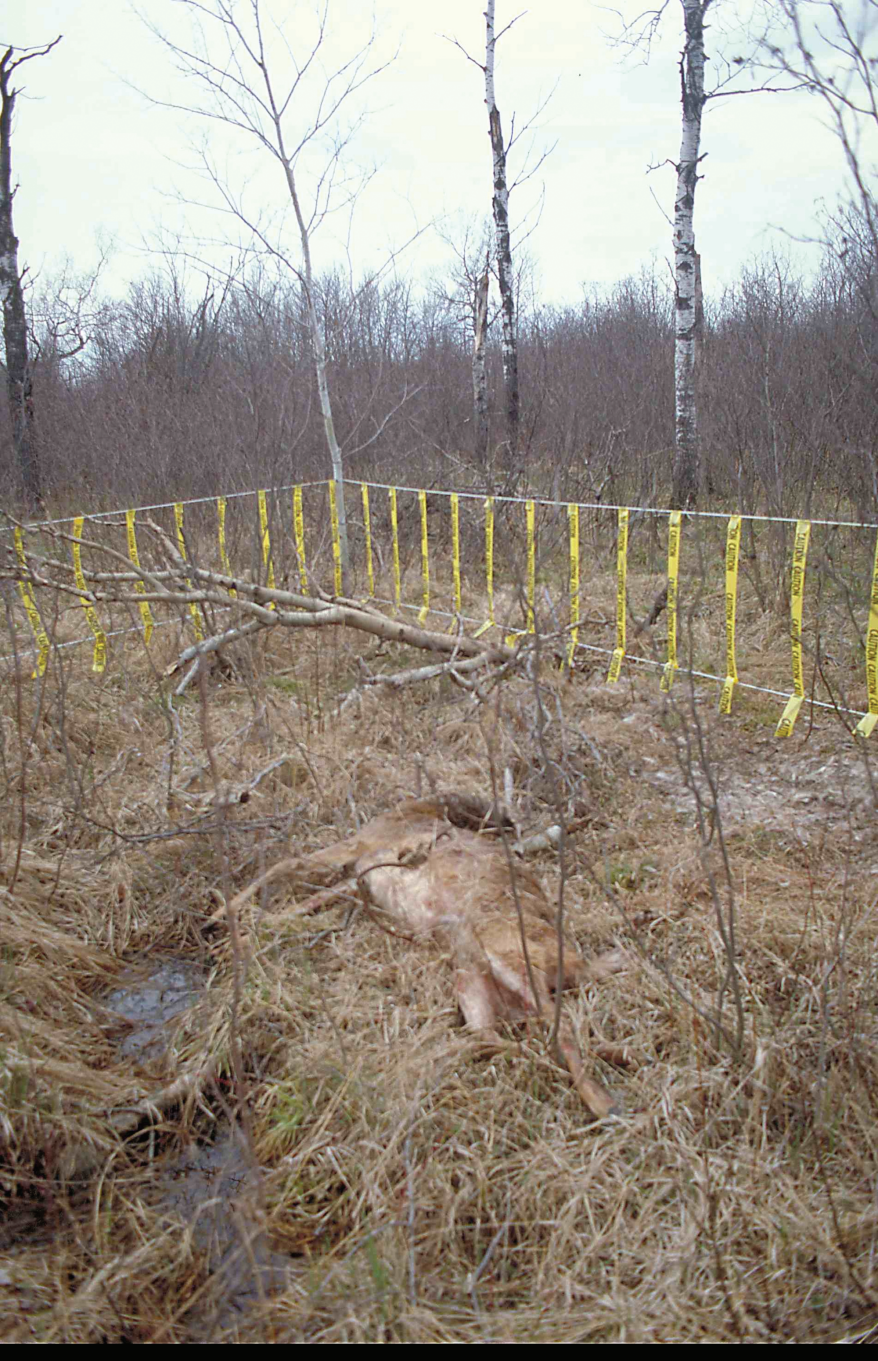
Fladry hung around a vehicle-killed deer carcass used in our earliest RCT (Shivik et al. 2003). Credit: A. Treves.

**Figure 5.**
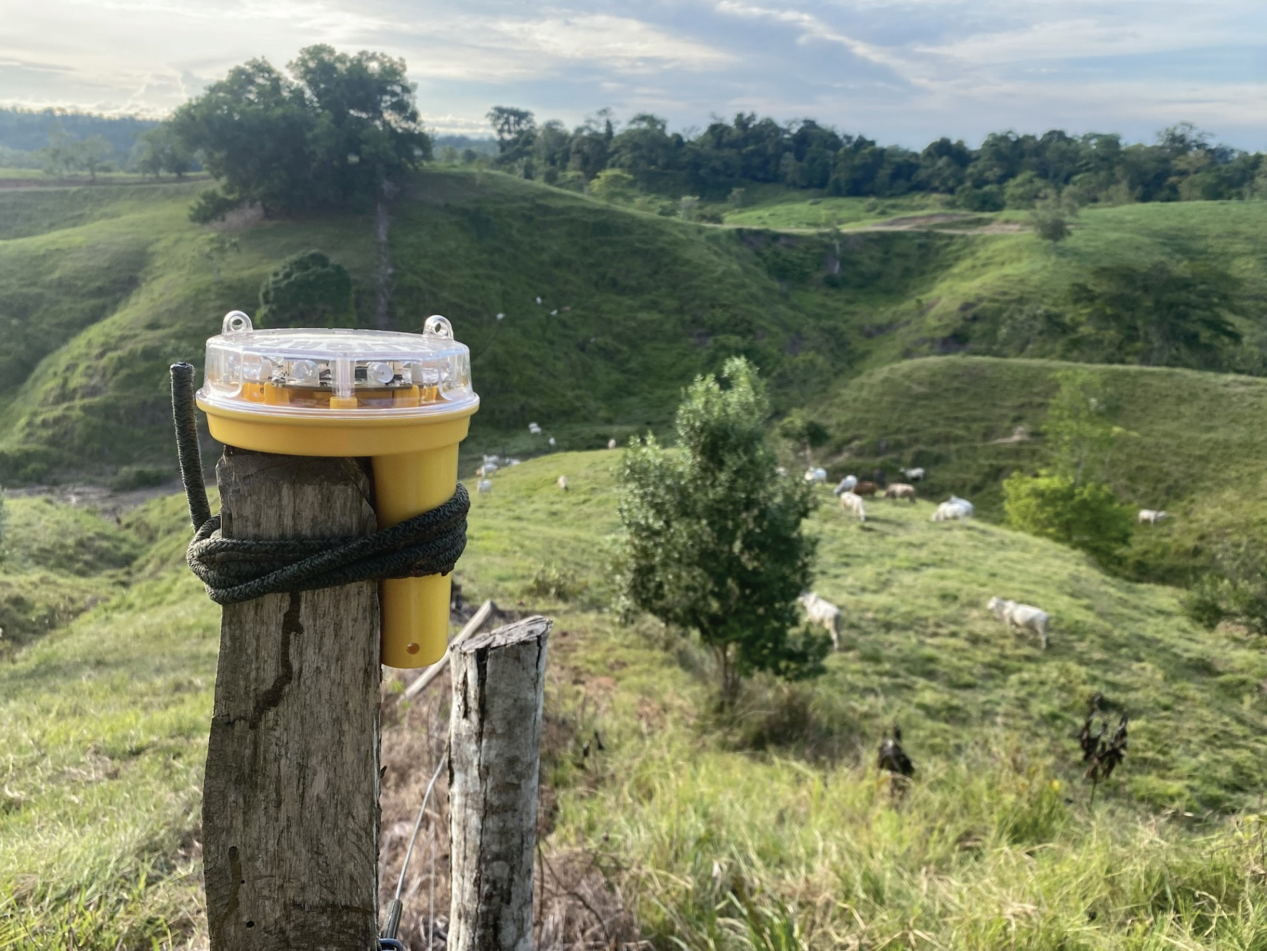
Foxlight mounted on a stationary fence post, Cimitarra, Colombia. Credit: A. Pineda-Guerrero.

The third RCT as yet not peer-reviewed but published (Fergus, 2020), evaluated the same deterrent lights as Ohrens et al., (2019a) combined with fladry a visual deterrent composed of flagging hung at regular intervals (Figures 4 and 5) to deter black bears, coyotes, and gray wolves from five herds of diverse livestock types in Wisconsin, United States (Fergus, 2020). Another RCT replicated (Fergus, 2020) in hopes of combining datasets (Hermanstorfer, 2023). Hermanstorfer (2023) aimed to protect five herds of various livestock from coyotes, pumas, black bears, foxes, and free-ranging, domestic cats in Colorado, United States, using the same deterrents (Figures 4 and 5). The latter two studies have been combined to glean more insight into indeterminate effects and the role of inter-subject variation in treatment effects (Fergus et al. 2023; see also Treves and Khorozyan, pre-print).

**Figure 6.**
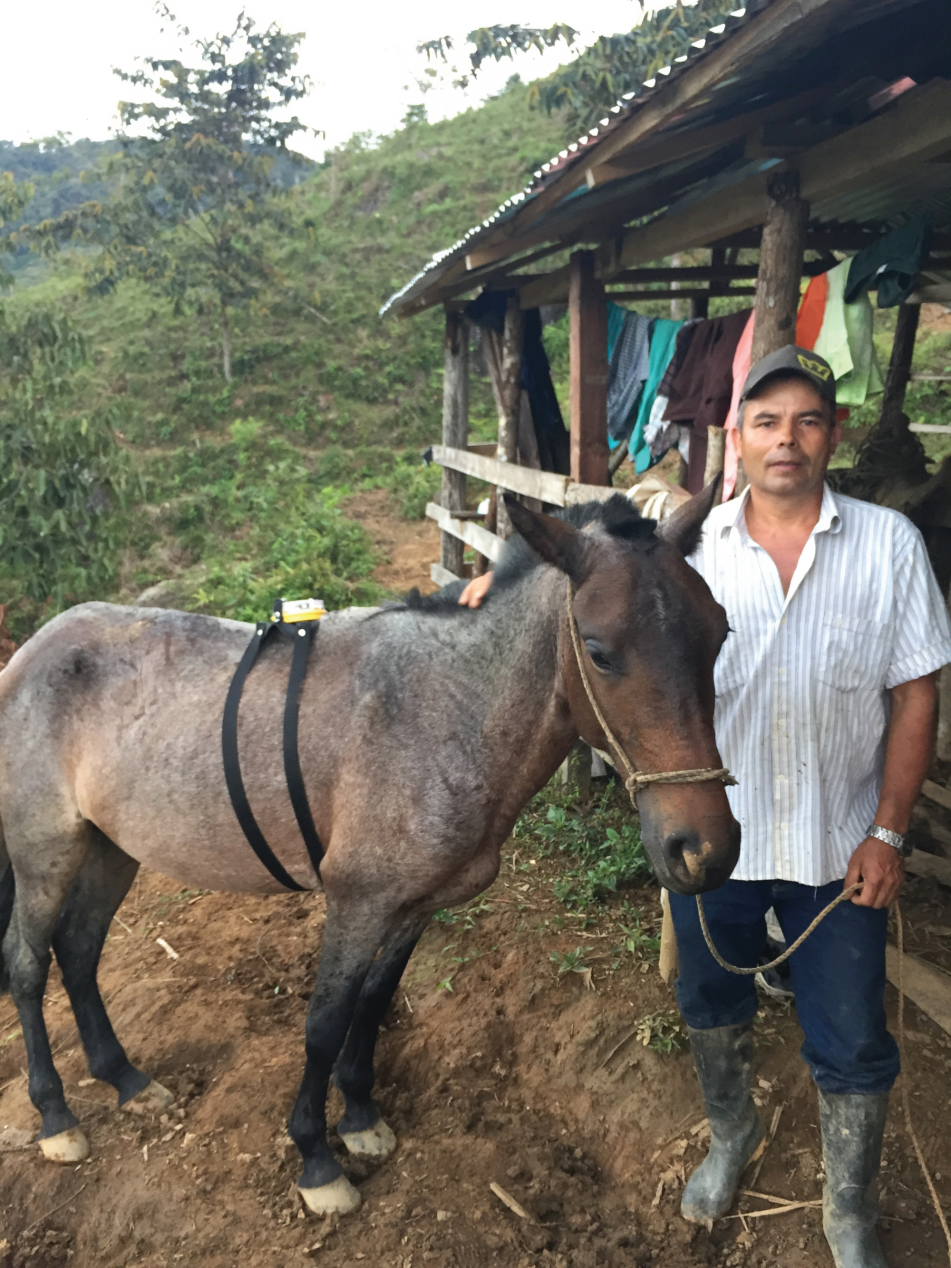
Foxlight mounted on the back of a domestic animal, San Luis, Colombia. Credit: A. Pineda-Guerrero.

**Figure 7.**
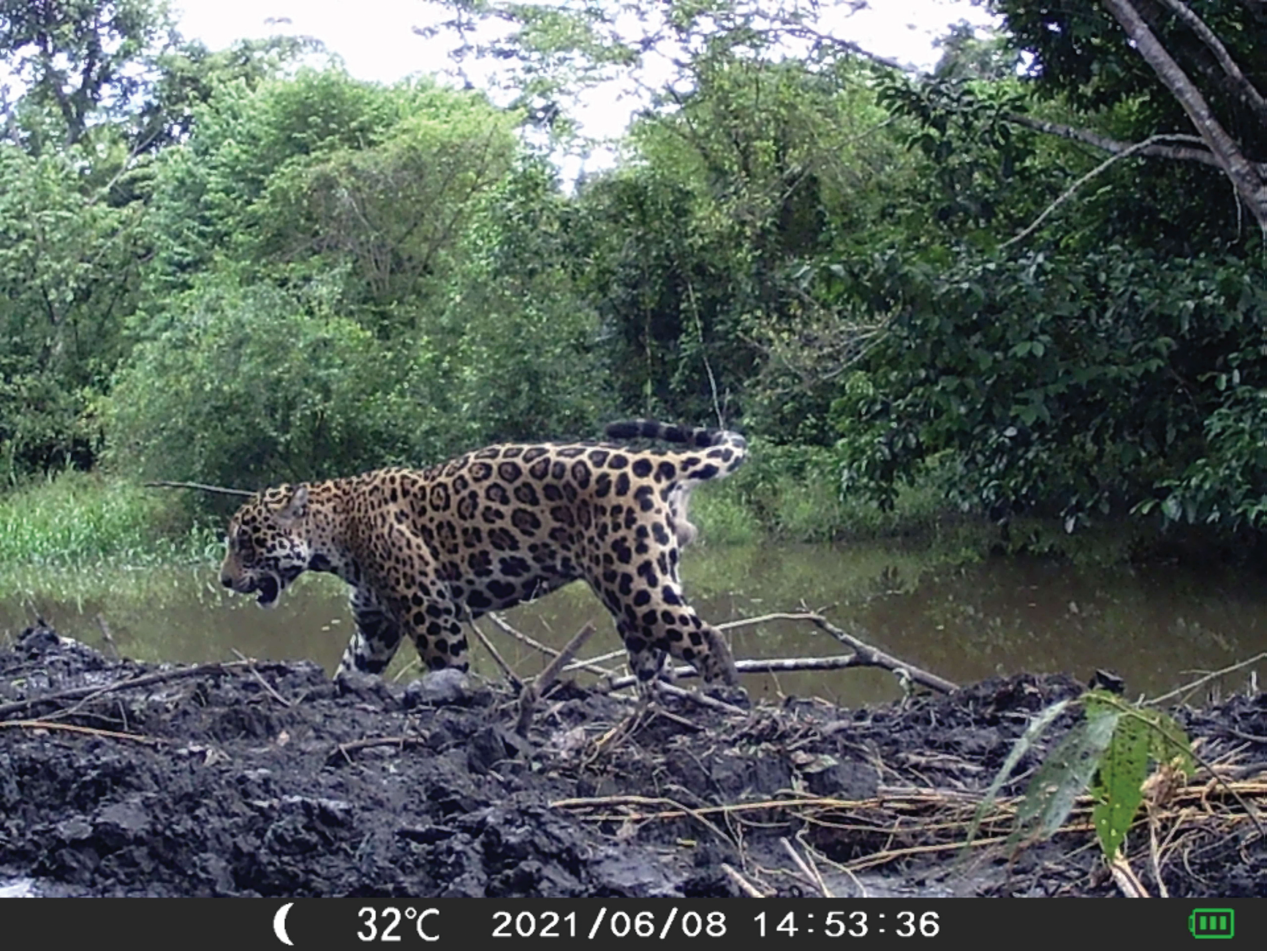
Jaguar at Cimitarra, Colombia. Credit A.A. PinedaGuerrero.

**Figure 8.**
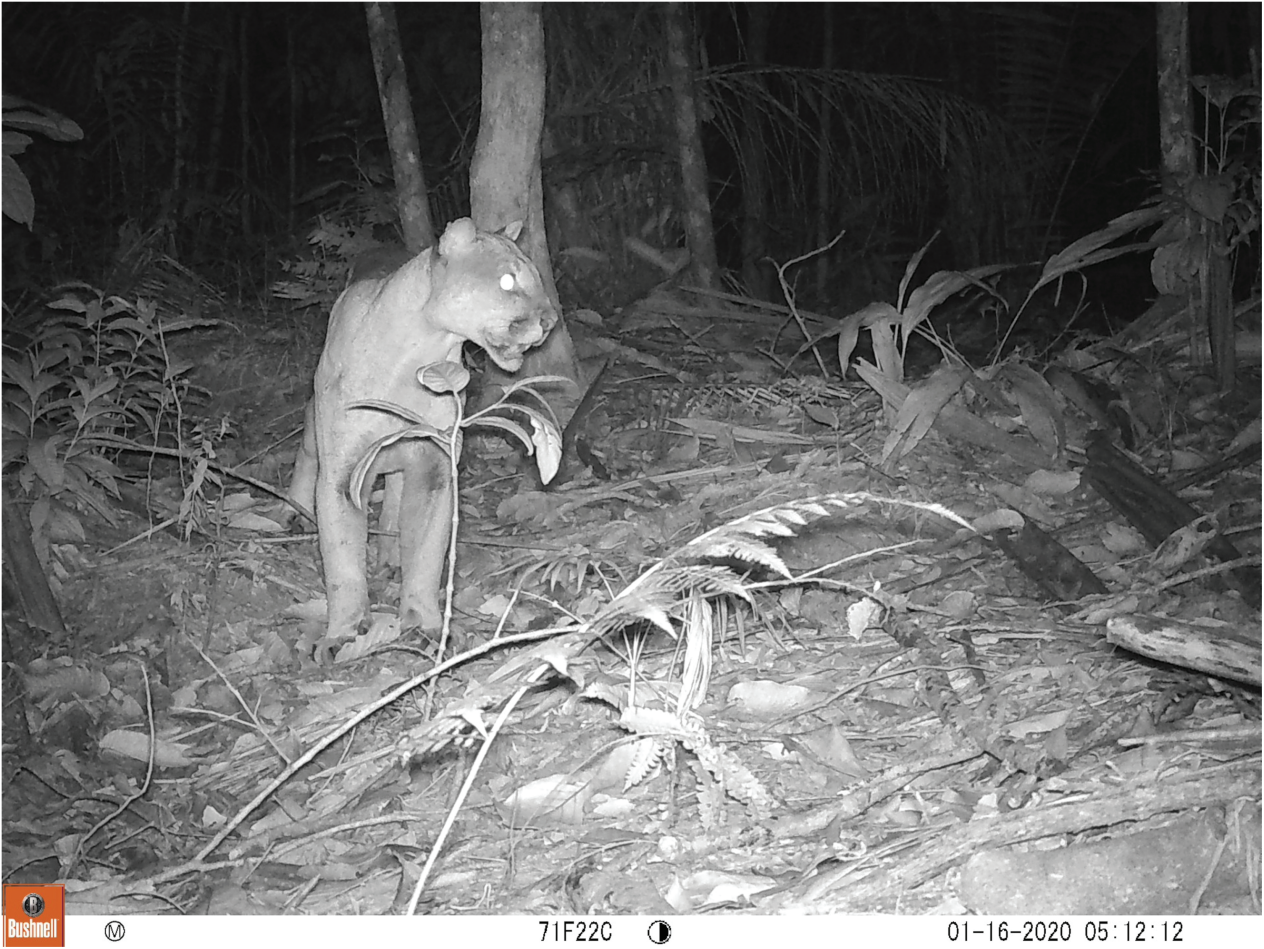
Puma at Cimitarra, Colombia. Credit A.A. Pineda-Guerrero.

Pineda-Guerrero’s dissertation RCT (Pineda Guerrero, 2023) aimed to protect 32 herds composed of a variety of livestock from approach by pumas and jaguars (*Panthera onca*) (Figures 5-8) at two Colombian forested sites, using stationary lights, as evaluated by Ohrens et al. (2019a), and a novel method never tested before: mobile deterrent lights (Figure 6). Her study offers the largest sample size subjected to our RCTs with crossover design (*n* = 25–32 depending on which effect was tested). Her study also offers insights into the use of true placebo controls versus inactive controls without placebo and an insight into taking farmers’ ideas about deterrents and implementing them within an RCT.

We present qualitative information and lessons we learned from field experiments in the following sections. Although some of the information may appear anecdotal because it arose from one or two studies, all of the qualitative information presented here and recommendations achieved consensus among the authors, unless otherwise noted and discussed below. We offer these lessons and insights as new hypothetical explanations or as methodological tips. We call repeatedly for replication of our findings and tests of our new hypotheses because we are aware that single studies or even the replicated ones we report, are not sufficient to guide regional policy or make confident recommendations to farmers. We hope, with these insights, we can help other researchers navigate randomized experimental trials with crossover designs in field conditions.

## Design Recommendations

Although we were not the first to complete RCTs under such conditions, our crossover designs and strict attention to avoiding sampling, treatment, measurement, and reporting biases (Treves et al., 2019) led to consistent methods that allow a systematic review of lessons learned.

The noninvasive methods used in our RCTs allowed us to obtain an exemption from the Institutional Animal Care and Use Committee at the University of Wisconsin-Madison. Also, our protocols fs 2016-1071-CP005, 2019-0194, and 2021-0923-CP002 protected human subjects.

*Recruitment—*Our general first step was to recruit owners or managers of livestock as participants. We use one or both individual recruitment interviews or group workshop-style recruitment methods (Treves et al. 2009). Some of the recruitment or attendance at workshops followed a snowball method or word-of-mouth between owners encouraging neighbors to attend and participate. We are aware this creates a potential bias due to non-independence in our attitudes dataset. Namely, word-of-mouth and snowball methods are likely to bring together participants of like mind and attitudes. Farms may also be managed similarly. Therefore, we emphasize the importance of replicating our findings in both RCTs and human dimensions. Once recruitment is complete, maintaining communication with participants becomes an ongoing task to establish trust. Participants may request to view photographs of wildlife on their farms and to stay informed about project updates.

All but one RCT (Louchouarn and Treves 2023) involved our team conducting semi-structured interviews before, during, and after implementation so the ‘during’ interview could measure response to treatment and control separately. All interviews measure attitudes to carnivores, perceived effectiveness of non-lethal methods, and satisfaction with the participatory experiments. Our studies ended with small sample sizes of attitudes, perforce, because recruitment to the RCT with crossover was our priority. We recommend more funding and more staff to expand the sample sizes of both arms of such research. Some of our data particularly (Pineda Guerrero, 2023) are the first of their kind to measure changes in participant attitudes during an RCT with crossover to evaluate predator control. Naturally, our findings demand replication before the findings shape policy or private investments.

*Sample sizes—*Our sample sizes of owners or managers and their herds dropped below our initial designs in four out of the five studies summarized above. Sample sizes declined at various points in the experiment because one owner stopped communicating (Ohrens); one quit in protest and lost or took our equipment (Pineda Guerrero); two herds could not be completely protected by fladry (Fergus, Hermanstorfer); the cameras around three herds malfunctioned (Pineda Guerrero); and one owner allowed two unprotected sheep to cross fences between forest and pasture, where wild felids killed them in the paddock holding subject cattle protected by our active deterrent lights (Pineda Guerrero, 2023). The rate of such ‘drop-outs’ ranged from 0% to 20% and drop-outs may only be partial, providing data for some analyses but not all. We are aware of only two owners who may have subverted the placebo control condition by turning on deterrent lights (Pineda Guerrero, 2023). One of those two owners also appeared on our cameras engaged in illegal activities and later lost or stole our cameras (Pineda Guerrero, 2023). In the latter study, we conducted a sensitivity analysis excluding those herds to avoid treatment bias (Pineda Guerrero, 2023). We acknowledge unforeseen circumstances (e.g., camera theft or participants turning on the lights when in placebo), can bias the treatment effect. However, we encourage researchers to openly share these experiences and approach data analysis in ways that minimize biases.

Owner’s attitudes to the non-lethal methods, carnivores, and coexistence with carnivores have been highly variable. The largest study (Pineda Guerrero, 2023) suggests increased tolerance for carnivores after the experiment and more positive attitudes about non-lethal methods, despite little or no evidence the methods protected their herds. We refer to this as a disparity between perceived effectiveness and functional effectiveness (Ohrens et al. 2019b). Also, improvements in attitude were more notable for owners or managers who began in control condition in phase 1 and then took on the treatment condition in phase 2 compared to owners who had the converse order of conditions (Pineda Guerrero, 2023). Hermanstorfer (2023) found mixed effects on attitudes with two out of five owners turning more negative to carnivores and three out of five remaining stable. He speculated that farm owners may be overall supportive of carnivore coexistence, but express acceptance until they are more comfortable with the researcher, which in turn allows them to be more cautious or intolerant in the later interviews. The findings of Pineda Guerrero (2023) and Hermanstorfer (2023) both may be accurate and might be incompatible, underlining the need for larger samples and replication with safeguards against non-independence in attitude data as mentioned earlier.

Although relationships with owners take a great deal of care and time invested in building trust and communications, we found individual owners easy to work with in general and more willing to engage in experimental studies including placebos, than organizations and agencies express in other public settings, consistent with related research showing representatives of interest groups espousing more extreme views than their constituents (Nilsen et al., 2007). For example, the U.S. Department of Agriculture Wildlife Services charged with managing agricultural damages has been explicitly adversarial, e.g., Western watersheds project et al. v. USDA Wildlife Services 2018. U.S. District Court for the District of Idaho. Therefore, we recommend personal contact for recruiting private farm owners.

*Farm animals and herds—*we conducted RCTs on alpacas, cattle, equids, llamas, and smaller stock including poultry, and sheep. Owners gathered animals in fenced, small pastures or large, unfenced habitats, and organized in homogeneous herds or herds of mixed animals that differed from herd to herd. Heterogeneous herds can produce uncertainty about mixed effects of non-lethal interventions. A lack of a statistically significant effect of a treatment may result from attraction to one subject herd and deterrence from another subject herd, which might reflect the differential attractiveness or vulnerability of different individuals in each subject herd (Fergus et al. 2023; Pineda Guerrero 2023). Nevertheless, homogeneous herds are not identical (Louchouarn and Treves, 2023). Demanding uniformity in subjects is an impossible ideal, and we mean impossible literally because individual traits of subject animals will always thwart efforts at uniformity. Crossover provides a more useful and efficient safeguard against variability between subjects. Heterogeneity does not by itself undermine the resulting inferences until research shows that the heterogeneity mimics or obscures a treatment effect (Ohrens et al.2019a; Treves et al. 2019; Pineda Guerrero 2023). We recommend larger samples when herd composition is heterogeneous. We also recommend preliminary evaluations of potentially confounding effects prior to evaluating a treatment or order effect in RCTs with crossover design (Pineda Guerrero, 2023).

Since our first RCT with live domestic animals, which experienced 47 losses of livestock (Ohrens et al. 2019a), losses have been very low: (Louchouarn & Treves 2023) verified 1 in the washout period, (Pineda Guerrero, 2023) verified 3 (2 on non-subjects), Fergus (2020) and Hermanstorfer (2023) reported zero each. Therefore, claims of rampant carnivore predation on livestock seem dubious in our regions.

We followed owner preferences for protecting their herds, while strictly randomizing treatment or control. Owners sometimes selected the deterrent methods or even invented a novel deployment. For example, in Pineda Guerrero (2023), owners suggested mounting deterrent lights to the backs of livestock animals. One owner had placed Nite Guard lights to the backs of domestic animals before the RCT with Foxlights. The deployment of Mobile Foxlights proved impossible for some animals (e.g., cattle, note Fergus (2020) also reported difficulties when cattle ate fladry.), but more readily accepted by others (e.g., some equids accepted the harnesses of mobile lights readily). Although we found no significant difference in effects of mobile lights versus stationary lights across herds (Pineda Guerrero, 2023), we suggest further research on mobile deterrents. Mobile deterrents can potentially overcome constraints imposed by large pastures, forested pastures, or scattered animals across pastures.

*Carnivores—*we always deployed trail cameras around subject herds, generally but not always oriented toward wild habitat not toward the pastures. Future RCT may involve owners and livestock managers in checking the proper functioning of trail cameras more often to prevent data loss. Two studies also conducted indirect sign surveys to estimate approaches by carnivores (Louchouarn and Treves, 2023; Ohrens et al. 2019a and in preparation).

Several of us suspect individual differences between carnivores or differences between species of carnivores may have influenced our results. The non-lethal methods we tested were intended to deter medium- and large-bodied felids and canids, in habitats that also contain bears or smaller felids and canids. For example, Ohrens et al. (2019a) reported a deterrent effect of Foxlights on pumas but a non-significant tendency to attract Andean foxes. His second still-unpublished study suggests pumas at that site were not deterred by the same lights.

Looking across the literature in our subfield of predator control, we see a major gap in understanding of the relationship between carnivore approaches to humans or domestic animals and the risk of actual attack. Because wild carnivores are elusive (i.e., they are almost always shy of people), they are very hard to detect by eyewitnesses (Chavez and Gese, 2006; Ordiz et al., 2013; Versluijs et al., 2021). Indirect methods such as telemetry, track and sign surveys, and trail cameras reveal that wild carnivores frequently approach (and leave) proximity to humans or domestic animals without any resulting attack. Our experiments with zero losses of farm animals could only infer with confidence about the frequencies of approaches to subject herds. Such approaches are frequent in some areas and less so in others in which we have run RCTs. These observations suggest two general recommendations.

First, larger samples and longer studies will be needed where approaches are rare (Mills et al., 2009). Response variables should be chosen with care and preferably include two types (approaches that may not reflect augmented risk for domestic animals) and actual hazards such as injuries or deaths of domestic animals. Indeed, regarding the former, methods for detecting approaches should prioritize close approaches rather than approaches at great distances because remote carnivores do not pose a danger to domestic animals until the potential predator closes the distance.

Second, individual variation in inclination to approach is likely to be a powerful variable. We hypothesize that an individual carnivore’s experience of exposure to deterrents, such as human lights, will modulate that individual’s reaction to a light deterrent. Habituation to deterrent stimuli has long been suspected (Linhart and Knowlton, 1975) and short-term effectiveness is a common feature of both non-lethal and lethal interventions (Khorozyan and Waltert, 2019). Mobile or random deterrents may slow habituation as might behavior-contingent deterrents (Shivik et al., 2003; Shivik, 2006). These variables associated with deterrent technology may interact in complex ways with the personalities of wild animals and their experience with the deterrents.

The issues of initial curiosity or initial deterrence followed by subsequent changes in individual carnivore behavior deserve more study. Louchouarn & Treves (2023) found increased presence of gray wolves during the first phase of the RCT in treatment herds, but not during the second phase, which occurred in the fall when wolves might range more widely. We hypothesized that curiosity by wolves could have attracted them to the new range riders, but this curiosity dissipated as wolves became accustomed to the new humans (Louchouarn & Treves 2023).

*Deterrents—*we emphasize caution and perpetual scrutiny of deterrent effectiveness using farmer-based monitoring methods and advanced analyses. A common issue in our experience is that non-lethal methods are only implemented on farms with previous attacks. To mitigate such bias from temporal and demographic autocorrelations (Treves and Khorozyan, pre-print; Treves and Chapman, 1996; Treves, 2001; Murtaugh, 2002; Stewart-Oaten, 2003), we recommend randomized designs and careful baseline measurements for comparison with treatment and order effects.

We agree with other authors that individual carnivores may habituate to deterrents and a mix of deterrent tools is safer than a single tool (citations above). For example, when two of us tested a nighttime light deterrent and a 24-h fladry deterrent, they split the data on carnivore approaches into daytime and nighttime periods to evaluate differences between single- and double-treatment conditions (Fergus, 2020; Fergus et al., 2023; Hermanstorfer 2023). Further work is needed to refine our understanding of multiple deterrents and even single interventions implemented against a backdrop of protective husbandry (Louchouarn & Treves 2023; Stone et al., 2017). The effect of non-lethal deterrents may depend on acclimatization by target carnivores. Ohrens’ first Chilean site (Ohrens et al. 2019a) was very remote with few lights and large distances between human structures, so pumas might have been unfamiliar with lights in that altiplano setting. In turn, perhaps Andean foxes were more accustomed to lights because they spent more time near human habitations or foxes avoided pumas, which made lights more attractive as in the hypothesis of human shields, e.g., Berger, (2007). By contrast, the temperate forest of Ohrens’ second site was more densely populated with both humans and pumas (in preparation). This was our first exposure to contrary and unexpected effects on one carnivore while finding the desired outcomes for another carnivore. Subsequently, Hall & Fleming (2021) reported elevated risk for piglets during moonlit nights and during an RCT using the same deterrent lights as Ohrens et al. (2019a). More complicated yet, Pineda Guerrero (2023) reported that jaguars initially approached the same deterrent lights at two of her study areas but a second field season revealed jaguars avoided the deterrent lights at the first study site when she repeated the experiment the next year. The attraction and deterrence of jaguars was not strongly statistically significant, so she hypothesized that curiosity can lead some individual carnivores to approach deterrents initially; and later deter them for reasons unknown. Taken together, our four RCTs using light deterrents of the Foxlights brand suggest these lights that randomly flash at night in three colors are unlikely to produce strong deterrent effects on carnivores unless those individuals are unaccustomed to lights. Also, the possibility of attracting two species of foxes and aiding their hunting (Ohrens et al.2019a; Hall and Fleming, 2021) should give users pause. Habituation is one potential explanation, but the role of human shields should be considered at the same time (see above).

Commentators often mistakenly ascribe the inadequacies of short-term effect and counter-productive results to non-lethal methods only. That would reflect a bias if no data support it. Indeed, by contrast lethal methods also experience variable durations of effect and counter-productive effects (Santiago-Avila et al., 2018; Khorozyan and Waltert, 2019, 2020; Treves et al., 2024). The field would benefit from more acknowledgment that all interventions have variable effects and benefit from understanding the causal mechanisms of such variability.

*Design—*we have had to vary nuances of our standard RCT with crossover, depending on conditions. For example, Louchouarn & Treves (2023) used a pseudo-control because owners refused the true placebo of no range rider present. They were happy to accept an RCT and a pseudo-control in which novice range riders, trained for only a short period, and were paired with an experienced range rider. We exploited this situation by maintaining the experienced range rider as the baseline pseudo-control condition in all subject herds and augmenting his work with one to two novice range riders for the treatment condition. This granted us new opportunities to test hypotheses about the number of range riders, frequency of their presence around herds, and level of experience as covariates. Given only one cow was killed during the wash-out period (between pseudo-control and treatment on the same herd), and given prior to our RCT there had been wolf and grizzly bear attacks on cattle in these same pastures, our results suggest range riders were protective. We predict that a higher frequency of range rider visits would be important for deterring grizzly bears.

Similarly, in the Colombian study, Pineda Guerrero (2023) evaluated dose effects measured by the number of light deterrents, two treatment permutations differing by their deployment as mobile or stationary, and two types of control (active placebo control or inactive control). Given her relatively large sample size (*n* = 25–32), Pineda Guerrero (2023) was able to evaluate multiple conditions while retaining some statistical power. Nevertheless, treatment effects were inconclusive (Pineda Guerrero, 2023).

## Effectiveness of Non-lethal Methods to Prevent Wild Carnivore Predation on Farm Animals

Overall, we repeat the common admonition that any intervention is an experiment and that intervenors would be wise to monitor the effectiveness rigorously. We are confident about two deployments of non-lethal methods to prevent predation on livestock. The first is the deployment of herders using low-stress livestock-handling techniques (Louchouarn & Treves 2023). The second is the deployment of light deterrents when wild carnivores are not already habituated to human lights with the caveats discussed above or when lights are paired with a day-time deterrent; however, both conditions can be highly variable across farms or herds (Ohrens et al. 2019a; Fergus et al. 2023). Our confidence in fladry is enhanced by independent replications from other RCTs and non-RCT studies (Davidson-Nelson and Gehring, 2010; Iliopoulos et al., 2019; Bruns et al. 2020) and references therein. Likewise, work in other countries is producing new insights into potentially effective non-lethal methods with larger sample sizes than have previously been achieved (Khorozyan et al., 2020; Radford et al., 2020). The field continues to follow the advice of Gehring et al. to design non-lethal methods so that domestic animal owners can install and maintain them independently. These seem promising trends and offer managers a way to find the triple-win for wild and domestic animals in addition to people.

## Conclusions

We recognize that any review and summary of evidence necessarily implies interpretations by the authors, with which other qualified experts may disagree. However, that disagreement must point to evidence as we have done, not simply dismiss or ignore the evidence we have marshaled. We are concerned about a tendency for individuals and organizations conducting applied research and management to ignore inconvenient evidence and dismiss research that strikes at fundamental assumptions about human-wildlife coexistence. Therefore, we encourage open scholarly debate centered on explicit methods, data, and inferences.

## About the Authors

**Adrian Treves** investigates ecology, scientific integrity, public trust principles, and agro-ecosystems where crops and domestic animals overlap carnivore habitat. Founder and Director of the Carnivore Coexistence Lab, and Professor of Environmental Studies at the University of Wisconsin-Madison, he earned his PhD at Harvard University. Beginning his career as a behavioral ecologist studying predator-prey interactions and infanticide, he pioneered in studies of vigilance before turning to carnivore-human-domestic animal interactions. The members of the Carnivore Coexistence Lab, CCL http://faculty.nelson.wisc.edu/treves/author peer-reviewed scientific articles and an equal number of other scientific papers, including ground-breaking estimates of the hazard and incidence of wolf-poaching, and modeling risk to predict human-carnivore conflict sites. Current work of the CCL focuses on experimental evidence for the landscape of fear, reintroduction of large carnivores, and responses of wild carnivores to human-associated stimuli.

**Abi R. Fergus** completed a master’s degree in Environment and Resources through the Nelson Institute at the University of Wisconsin Madison. During graduate school, Abi researched the effectiveness of the deterrents fladry and Foxlights® at reducing carnivore visits to six livestock farms within the Bad River Tribe’s Ma’iingan (Gray Wolf; *Canis lupus*) buffer zone. Abi utilized tracking skills to target camera traps and deterrents where carnivore species and the deer they often follow seemed to be approaching livestock areas on farms. Abi is now certified at the Tracker 3 level in the international CyberTracker evaluation system. Abi has utilized these tracking skills to monitor the wolf packs on the Bad River Reservation as the Tribe’s wildlife specialist. Abi has also worked as a contractor for the Lac Courte Oreilles Tribe to train the wildlife biologist in wolf howl surveys, tracking, and camera trapping to study the Reservation wolf packs.

**Samuel J. Hermanstorfer** was awarded his masters of science at the Nelson Institute for Environmental Studies at the University of Wisconsin—Madison. His research investigated carnivore coexistence in western Colorado through crossover-design, randomized-controlled trials of non-lethal deterrent devices, and interviews with participating livestock owners. Professionally, Samuel spent a summer as a coastal bird research intern with The Wetlands Institute (Stone Harbor, NJ), where he studied Fish Crow behavior. He also worked as a zookeeper intern at Brookfield Zoo (Brookfield, IL). Sam currently works for the Wisconsin Department of Natural Resources in Milwaukee, WI.

**Naomi X. Louchouarn** earned her PhD and currently works as a research scientist at the Nelson Institute at the University of Wisconsin-Madison, USA. She received her Bachelor’s of Science from McGill University with dual minors in wildlife biology and applied ecology. She then received a Master’s of Environmental Science and Management at UC-Santa Barbara’s Bren School where she focused on conservation planning. Her PhD research has focused on examining the effectiveness of tools and policies to mitigate human-carnivore conflict in North America. She has also explored and published peer-reviewed papers about what makes high-quality carnivore science.

**Omar Ohrens** is a Chilean agronomist from the Catholic University of Chile. After his professional degree, he worked as an associate researcher at the Fauna Australis Wildlife Laboratory working on several wildlife conservation projects throughout the country. He then completed a master and doctorate degree at the University of Wisconsin-Madison at the Carnivore Coexistence Lab. His graduate research focused on the conservation of carnivores, including pumas, and human-carnivore coexistence in Chile. His research has been funded by the US Fish and Wildlife Service-Wildlife Without Borders conservation program and the National Geographic Society-Conservation Trust. He is currently a Conservation Scientist for the NGO Panthera, Puma program, focusing on puma conservation in Latin America. Specifically, his work focuses on range-wide assessments of pumas across Latin America, puma-human conflict and coexistence, and facilitating and expanding Panthera’s puma conservation strategy in Latin America.

**Alicia Alexandra Pineda-Guerrero** is a Colombian biologist. After earning her bachelor’s degree, from Javeriana University she worked with the NGO ProCAT Colombia, where she contributed to conservation projects in Colombia and Costa Rica. She has conducted research projects in collaboration with government environmental agencies, NGOs, and local communities in Colombia. Since 2015, she has been a member of the Carnivore Coexistence Lab, having joined during her master’s at University of Wisconsin-Madison. In October 2023, she successfully defended her dissertation. Her doctoral research focused on the functional and perceived effectiveness of a non-lethal method to prevent puma and jaguar attacks in two areas in Colombia. She also conducted a longitudinal study measuring changes in attitudes of participants in RCTs. Her research interests include human-carnivore interactions, carnivore ecology, the human dimensions of conflict and applied research to inform decision making.
